# Bruton’s Tyrosine Kinase, a Component of B Cell Signaling Pathways, Has Multiple Roles in the Pathogenesis of Lupus

**DOI:** 10.3389/fimmu.2017.01986

**Published:** 2018-01-22

**Authors:** Anne B. Satterthwaite

**Affiliations:** ^1^Department of Internal Medicine, The University of Texas Southwestern Medical Center, Dallas, TX, United States; ^2^Department of Immunology, The University of Texas Southwestern Medical Center, Dallas, TX, United States

**Keywords:** Bruton’s tyrosine kinase, lupus, autoantibody, B cell, plasma cell, Lyn

## Abstract

Systemic lupus erythematosus (SLE) is an autoimmune disease characterized by the loss of adaptive immune tolerance to nucleic acid-containing antigens. The resulting autoantibodies form immune complexes that promote inflammation and tissue damage. Defining the signals that drive pathogenic autoantibody production is an important step in the development of more targeted therapeutic approaches for lupus, which is currently treated primarily with non-specific immunosuppression. Here, we review the contribution of Bruton’s tyrosine kinase (Btk), a component of B and myeloid cell signaling pathways, to disease in murine lupus models. Both gain- and loss-of-function genetic studies have revealed that Btk plays multiple roles in the production of autoantibodies. These include promoting the activation, plasma cell differentiation, and class switching of autoreactive B cells. Small molecule inhibitors of Btk are effective at reducing autoantibody levels, B cell activation, and kidney damage in several lupus models. These studies suggest that Btk may promote end-organ damage both by facilitating the production of autoantibodies and by mediating the inflammatory response of myeloid cells to these immune complexes. While Btk has not been associated with SLE in GWAS studies, SLE B cells display signaling defects in components both upstream and downstream of Btk consistent with enhanced activation of Btk signaling pathways. Taken together, these observations indicate that limiting Btk activity is critical for maintaining B cell tolerance and preventing the development of autoimmune disease. Btk inhibitors, generally well-tolerated and approved to treat B cell malignancy, may thus be a useful therapeutic approach for SLE.

## Introduction

The development of a B cell repertoire capable of secreting antibodies against a wide range of foreign antigens is crucial for effective immune responses. However, the processes that generate this diversity also result in the production of self-reactive B cells that, if not kept in check, can be pathogenic and lead to autoimmune disease. Systemic lupus erythematosus (SLE) is an autoimmune disease characterized by autoantibodies against nuclear antigens. These autoantibodies promote disease pathogenesis by forming immune complexes that deposit in and damage tissues and synergize with innate immune defects to sustain pro-inflammatory feed-forward loops (1). Autoantibodies arise prior to the development of overt clinical symptoms (2), suggesting that loss of B cell tolerance is an important initiating event in lupus. Understanding the signaling pathways that mediate autoantibody production may reveal new therapeutic targets for SLE, currently treated primarily by non-specific immunosuppression. Here, we review the contribution of Bruton’s tyrosine kinase (Btk) to lupus, with a focus on its role in B cells.

## Btk in B Cell Development and Activation

Bruton’s tyrosine kinase is a Tec family tyrosine kinase expressed in B and myeloid cells. It was first identified as the genetic defect in the primary immunodeficiency X-linked agammaglobulinemia (XLA) (3, 4). XLA patients have a block in B cell development at the pre-B stage and a paucity of circulating B cells and immunoglobulin (5). A mutation in the pleckstrin homology (PH) domain of Btk was subsequently found in X-linked immunodeficient (xid) mice (6, 7), which also have a B cell immunodeficiency, although milder than that of XLA patients. Btk-deficient mice phenocopy xid mice, with a block in B cell development at the immature stage, reduced peritoneal B1a cells, and impaired response to T-independent type II antigens (8–10).

Bruton’s tyrosine kinase is an important proximal component of B cell receptor (BCR) signaling pathways. Upon BCR engagement, the Btk PH domain binds to PIP3, a signaling intermediate generated by PI3 kinase (PI3K), thus localizing Btk to the plasma membrane (11–14). This facilitates its phosphorylation and activation by Src kinases (12, 13, 15) and promotes access to its substrates. The most well described of these is PLCγ2, which is activated after phosphorylation by Btk (16, 17) leading to increased Ca^++^ flux (14, 16, 18) and activation of NF-κB (19–21). BCR-induced proliferation and survival are impaired in the absence of Btk (9, 22–24). Btk is also required for toll-like receptor (TLR)-induced IL-10 expression by B cells (25, 26), and for synergy between the BCR and TLRs in enhancing IL-6 expression (27). Integrin-mediated adhesion of B lineage cells (28) and their response to chemokines, such as SDF-1 (29, 30), are also controlled by Btk.

## Btk and Mouse Lupus Models

### Btk Is Required for Autoantibody Production and Pathogenesis in Many Lupus Models

The xid mutation has long been known to reduce autoantibody levels in several murine lupus models, including NZB × NZW (31), BXSB (32), MRL.lpr (33), motheaten (34), and Gld (35). Renal disease was also prevented and survival improved by the xid mutation in NZB × NZW (31), BXSB (32), and MRL.lpr (33) mice.

The subsequent finding that Btk is a B cell signaling molecule suggested that enhanced B cell activation through Btk underlies autoantibody production in lupus models. This was tested using mice lacking B cell inhibitory signaling molecules. BCR signaling is normally limited by inhibitory receptors such as FcγRIIb, CD22, SiglecG, PIR-B, and CD72. The ITIMs of these receptors are phosphorylated by the tyrosine kinase Lyn, which results in the recruitment and activation of inhibitory phosphatases, such as SHIP and SHP-1 [reviewed in Ref. (36–38)]. B cell-specific deletion of Lyn, SHIP, or SHP-1 leads to B cell hyper-responsiveness and lupus-like autoimmune disease in mice (39–41). Mutations in inhibitory receptors result in milder autoimmunity, likely due to some degree of redundancy among them (42–47). Several of these inhibitory pathways target activating signals mediated by Btk (14, 16, 48).

Either the xid mutation (49) or Btk-deficiency (50–52) ameliorates the autoimmune phenotype of Lyn^−/−^ mice. Similarly, Btk is required for autoantibodies in FcγRIIb^−/−^.Yaa mice, which lack the inhibitory receptor FcγRIIb and also have enhanced TLR7 signaling (53). One caveat to these studies is that the reduction in mature B cells in xid and Btk^−/−^ mice is exacerbated in the absence of Lyn. To circumvent this defect, a transgene expressing a low level of Btk in B cells (Btk^lo^) (22) was crossed to Lyn^−/−^Btk^−/−^ mice (50–52). This normalized mature follicular B cell numbers to that of Lyn^−/−^ mice. However, Lyn^−/−^Btk^lo^ mice failed to produce autoantibodies or develop kidney damage, indicating that Btk signaling in mature B cells, rather than simply effects of Btk on B cell development, is critical for autoimmunity.

The autoimmunity caused by loss of Lyn-dependent inhibitory signaling is likely mediated by heightened Btk responses, as it is mitigated by reducing Btk dosage. This is supported by gain-of-function studies in which either a constitutively active form of Btk [which carries a PH domain mutation that enhances Btk membrane localization (54)] or wild-type Btk were overexpressed in the B lineage (55–57). In both cases, autoimmunity ensued. Limiting Btk signal strength in B cells is thus critical to prevent the loss of B cell tolerance.

### Multiple Functions of Btk Contribute to Autoantibody Production

#### Autoreactive B Cells Are Present in the Periphery in the Absence of Btk

Developing B cells are subjected to a central tolerance checkpoint at the immature B stage in the bone marrow. Those cells that express autoreactive receptors undergo receptor editing, rearranging a new Ig light chain to change their specificity. Cells that remain self-reactive after editing are deleted by apoptosis or rendered anergic. Autoreactive cells that escape are kept in check by peripheral tolerance mechanisms.

Taken together, the following observations suggest that Btk acts primarily in the periphery, rather than the bone marrow, to drive a loss of B cell tolerance. In Btk^−/−^ mice carrying an anti-DNA Ig transgene, anti-DNA B cells are present in the periphery but do not produce antibodies *in vivo* (26). Single cell repertoire analysis of new emigrant B cells (recently arrived in the periphery from the bone marrow) from XLA patients revealed a higher frequency of autoreactive B cells than in healthy controls (58). This indicates that Btk signaling may actually promote central tolerance, and that Btk-deficiency does not abrogate autoimmunity simply by preventing autoreactive B cells from reaching the periphery. Furthermore, immunoglobulin transgenic mouse models and analysis of XLA patient B cell repertoires have shown that receptor editing is independent of Btk (58–60). A role for Btk in the loss of peripheral B cell tolerance is highlighted by both loss-of-function and overexpression studies. Btk is required for autoimmunity in Lyn^−/−^ mice (49–52), which have intact central tolerance but develop autoantibodies due to a breach of peripheral tolerance (61, 62). Mice overexpressing Btk in mature B cells and myeloid cells, but not at earlier stages of B cell development in the bone marrow, develop autoimmunity (56).

#### Btk Contributes to Autoantibody Production beyond Its Role in Initial B Cell Activation

How does Btk signaling in the periphery drive autoantibody production? The role of Btk in the initial activation of BCR signals likely contributes, as residual B cells in Lyn^−/−^ xid and Lyn^−/−^Btk^−/−^ mice proliferate poorly in response to anti-IgM (49, 50). However, Lyn^−/−^Btk^lo^ B cells, like Lyn^−/−^ B cells, have increased proliferative response to BCR engagement (50, 52), suggesting that in the absence of Lyn-mediated inhibitory signaling, low levels of Btk are able to transmit some aspects of BCR signals efficiently. However, Lyn^−/−^Btk^lo^ mice do not develop autoantibodies or autoimmune disease (51, 52). Similarly, although Btk-deficient anti-DNA transgenic mice do not produce autoantibodies (26), Btk is not required for B cells from these mice or from AM14 rheumatoid factor (RF) transgenic mice to proliferate in response to nucleic acid-containing antigens (26, 63). Such autoantigens, common in lupus, activate B cells *via* both the BCR and nucleic acid-sensing TLRs (1). Thus, Btk has additional functions beyond transmitting proliferative signals from the BCR and TLRs that promote the loss of B cell tolerance.

#### Btk Drives Plasma Cell (PC) Accumulation

Accumulation of antibody-secreting PCs in the periphery is characteristic of SLE patients (64, 65) and murine lupus models, including Lyn^−/−^ mice (51, 66–74). A subset of inactive SLE patients demonstrate a PC-focused gene expression profile in their B cells, indicating that some patients may have an intrinsic predisposition to inappropriate B cell terminal differentiation (75). Btk is required for PC accumulation, as the increased PC frequency observed in Lyn^−/−^ mice is normalized in Lyn^−/−^Btk^lo^ mice (51). This is likely due to enhanced Btk signaling in B cells, since B cell-specific overexpression of either constitutively active or wild-type Btk also results in elevated splenic PCs (55, 56).

Activating signals by Btk and inhibitory signals by Lyn converge on the transcription factor Ets1 (76). Ets1 is expressed in resting B cells and limits PC differentiation by inhibiting the activity of Blimp1 (77), a master PC transcription factor. Ets1^−/−^ mice accumulate PCs and develop lupus-like autoimmunity, similar to Lyn^−/−^ mice (71). Ets1 levels are significantly reduced in B cells from mice deficient in Lyn or the inhibitory signaling components SHP-1 or CD22 plus SiglecG, but are normalized in Lyn^−/−^Btk^lo^ B cells (76). Restoration of Ets1 expression to Lyn^−/−^ or SHP-1^−/−^ B cells prevents excessive B cell differentiation *in vitro* (76). These observations indicate that autoreactive PCs accumulate in Lyn^−/−^ mice at least in part because of excessive downregulation of Ets1 by Btk. This is likely an exacerbation of a normal process, as BCR signaling downregulates Ets1 in wild-type B cells in a Btk-dependent manner (76). TLR signaling also downregulates Ets1 in wild-type B cells, and synergizes with BCR signaling to do so (76). In contrast, failure to downregulate Ets1 in response to Btk signals results in decreased steady state PC levels, as demonstrated by the ability of Ets1-deficiency to rescue the reduction in IgM antibody-secreting cells that occurs in Btk^−/−^ mice (78). Thus, a continuum of Btk signaling to Ets1 controls PC frequencies, and can result in autoimmunity, normal responses, or immunodeficiency depending on the signal strength (78).

#### Btk Promotes Class Switching of Autoreactive B Cells

Class switching to IgG is required for autoantibodies to be pathogenic (79). In Btk^lo^ mice carrying the 56R anti-DNA immunoglobulin transgene, anti-DNA IgM, but not IgG, is produced (26). Thus, Btk also promotes class switching of autoreactive B cells separate from its role in their initial activation and terminal differentiation.

Several functions of Btk contribute to this process. Expression of the class switching factors AID and T-bet is reduced in TLR-stimulated Btk^−/−^ and Btk^lo^ B cells relative to wild-type cells (26). Btk likely also plays an indirect role in class switching. IL-6 is required for IgG autoantibodies and autoimmune disease in Lyn^−/−^ mice (51, 70, 80). Lyn^−/−^Btk^lo^ mice have decreased serum IL-6 levels and a reduced frequency of myeloid cells expressing IL-6 in response to LPS compared to Lyn^−/−^ mice (51). B cell-derived IL-6 is also increased in Lyn^−/−^ mice (70), and is required in other models for the formation of autoreactive germinal centers, in which class switching occurs (81, 82). Btk is required for the upregulation of IL-6 in B cells in response to synergistic BCR and TLR9 signaling (27), and B cells overexpressing Btk express more IL-6 (57). Btk also promotes expression of IL-21, a Tfh-derived cytokine, in Lyn^−/−^ mice. This likely occurs *via* IL-6 as splenocytes from both Lyn^−/−^Btk^lo^ and Lyn^−/−^IL6^−/−^ mice have reduced expression of IL-21 mRNA compared to Lyn^−/−^ mice (80). Furthermore, Btk overexpression in B cells results in increased Tfh cells and IFNγ-producing T cells (57), which are important for autoreactive germinal centers and pathogenic autoantibodies (83–86).

#### Btk and Innate-Like B Cells

Bruton’s tyrosine kinase is expressed in B1a and marginal zone (MZ) B cells. These innate-like B cells may have both pathogenic and protective roles in autoimmune disease. The relative importance of Btk in these specific roles is not clear.

B1a cells are found predominantly in the peritoneal cavity, have a repertoire enriched in polyreactivity (87, 88), and are increased in several lupus models (41, 42, 88–90). Whether they are elevated in Lyn^−/−^ mice is controversial (52, 72, 91, 92). They secrete protective IgM autoantibodies (93) and the anti-inflammatory cytokine IL-10 (94, 95). In some cases they do not contribute to pathogenic autoantibodies (96), but they can produce IgG autoantibodies and interact with T cells in a pro-inflammatory manner in some lupus models (88, 97, 98). B1a cells are reduced in Lyn^−/−^Btk^lo^ mice (52) and increased in mice expressing constitutively active Btk (55), and Btk is required for their expression of IL-10 (25).

Marginal zone B cells are also enriched in autoreactivity (99–101). Whether they contribute to pathogenic autoantibodies in lupus is model-dependent (71, 101–107), and may be modulated indirectly by alterations in splenic architecture in some strains. Btk is not required for MZ B cell development, but it controls the positive selection of particular B cell specificities into the MZ compartment (108). How this affects autoimmunity is not clear. Skewing of autoreactive B cells to the MZ is promoted by Btk in the 56R anti-DNA immunoglobulin transgenic model (26) and in NOD mice (109), a model of type I diabetes, but RF B cells carrying the xid mutation are enriched in the MZ relative to their wild-type counterparts (63).

### Btk Inhibitors Are Effective in Mouse Lupus Models

The genetic evidence described above suggests that small molecule inhibitors of Btk could be an effective therapy for SLE. Preclinical studies with several inhibitors in multiple mouse models suggest that this may indeed be the case (Table 1) (110–117). Kidney damage was ameliorated in all cases and survival increased when measured. Btk inhibitors diminished B cell activity, as measured by reduced CD69 expression, PC frequencies, and/or germinal center B cell frequencies. Autoantibodies were also decreased, although in some cases not all specificities or isotypes were affected. Interestingly, kidney damage was prevented even in the few situations where IgG autoantibodies were not significantly reduced. This indicates that Btk has roles in lupus pathogenesis beyond its contribution to the loss of B cell tolerance. Btk inhibitors were effective in anti-GBM models of kidney disease, which measure only the effector phase of kidney inflammation and damage and do not depend on autoantibody production (113, 117). Btk inhibitors impair pro-inflammatory FcR responses of myeloid cells *in vitro* (111–113, 116–119), suggesting that Btk-dependent effector functions of myeloid cells may contribute to end-organ damage *in vivo*. However, Btk-deficiency in myeloid cells can have pro- or anti-inflammatory effects dependent on cell type and stimulus (120–135), and off-target effects of inhibitors cannot be ruled out (136). For instance, the Btk inhibitor ibrutinib also inhibits Itk (137), a related Tec kinase which has important functions in T cells. Further studies of the relative roles of B and myeloid cell-expressed Btk in lupus pathogenesis would be facilitated by the development of cell type-specific Btk knockout mice.

**Table 1:** Effects of Bruton’s tyrosine kinase (Btk) inhibitors on B cell activation and end-organ damage in murine lupus models.

Model	Inhibitor	Autoantibodies	B cell activation	End-organ damage	Survival	Reference
MRL.lpr	PCI-32765 (ibrutinib)	IgG reduced but not significantly		Reduced (kidney)		(110)
MRL.lpr	HM71224	IgG reduced	Reduced (CD69)	Reduced (kidney, skin lesions)		(111)
NZBxNZW	HM71224	IgG reduced but not significantly	Reduced [CD69, plasma cells (PCs)]	Reduced (kidney)	Increased	(111)
NZBxNZW	RN486	IgM unchanged, IgG reduced	Reduced (CD69, PCs)	Reduced (kidney)		(112)
NZBxNZW	PF-06250112	IgG reduced	Reduced (PCs, GCs)	Reduced (kidney)		(113)
NZBxNZW	G-744	Total reduced	Reduced (GCs)	Reduced (kidney)	Increased	(114)
IFN-enhanced NZBxNZW	G-744	Total ANA reduced, anti-dsDNA unchanged	Reduced (proliferation, PCs, GCs)	Reduced (kidney)	Increased	(114)
Sle1.Sle3	PCI-32765 (ibrutinib)	IgM and IgG reduced	Reduced (CD69, PCs)	Reduced (kidney)		(115)
BXSB.Yaa	M7583	Total reduced	Reduced (CD69, PCs)	Reduced (kidney)	Increased	(116)
DBA/Pristane	M7583	Total reduced except anti-SmRNP	Reduced (PCs), increased (CD69)	Reduced (arthritis)		(116)
Anti-GBM	PF-06250112			Reduced (kidney)		(113)
Anti-GBM	BI-BTK-1			Reduced (kidney)		(117)

## Btk in Human Autoimmunity

While polymorphisms in Btk have not been identified in GWAS studies of SLE or other autoimmune diseases, several lines of evidence suggest that increased Btk activity may be associated with autoantibody production in humans. Increased Btk expression and phosphorylation was observed in B cells from rheumatoid arthritis patients (138, 139), correlating with RF antibodies among RF-positive patients (139) and enriched in anti-citrullinated protein antibody-positive patients (138). Similarly, increased Btk expression and phosphorylation correlated with RF antibodies in Sjogren’s syndrome patients (138). The frequency of Btk^+^ cells in the peripheral blood of SLE patients has been reported to correlate with disease activity, anti-dsDNA antibodies, proteinuria, and C3 levels (140), but whether this reflects changes in Btk signaling or cell subset distribution is unclear.

Several SLE-associated signaling defects and polymorphisms likely result in increased activity of Btk signaling pathways in B cells. Reduced expression of PTEN, which counteracts PI3K, has been observed in human lupus B cells (141). Btk activation and function require the binding of its PH domain to the product of PI3K in the plasma membrane (11–14), and PTEN haploinsufficiency enhances the efficiency of Btk signaling in mice (142). The autoimmune phenotype in Lyn^−/−^ mice is mediated by excessive Btk activity in mice (49–52, 76, 80). Polymorphisms in Lyn are associated with SLE (143, 144), and reduced Lyn expression is observed in B cells from a subset of SLE patients (145–147). Expression of CSK, an inhibitor of Lyn, is increased by an SLE-associated polymorphism in the CSK gene. B cells carrying this SNP have reduced Lyn activity and increased responses to BCR signaling (148). Finally, several polymorphisms in Ets1 are associated with SLE, and Ets1 expression is reduced in PBMCs from SLE patients [reviewed in Ref. (149)]. Btk promotes the accumulation of autoreactive PCs in Lyn^−/−^ mice by downregulating Ets1 (76).

## Conclusion

**Figure 1:**
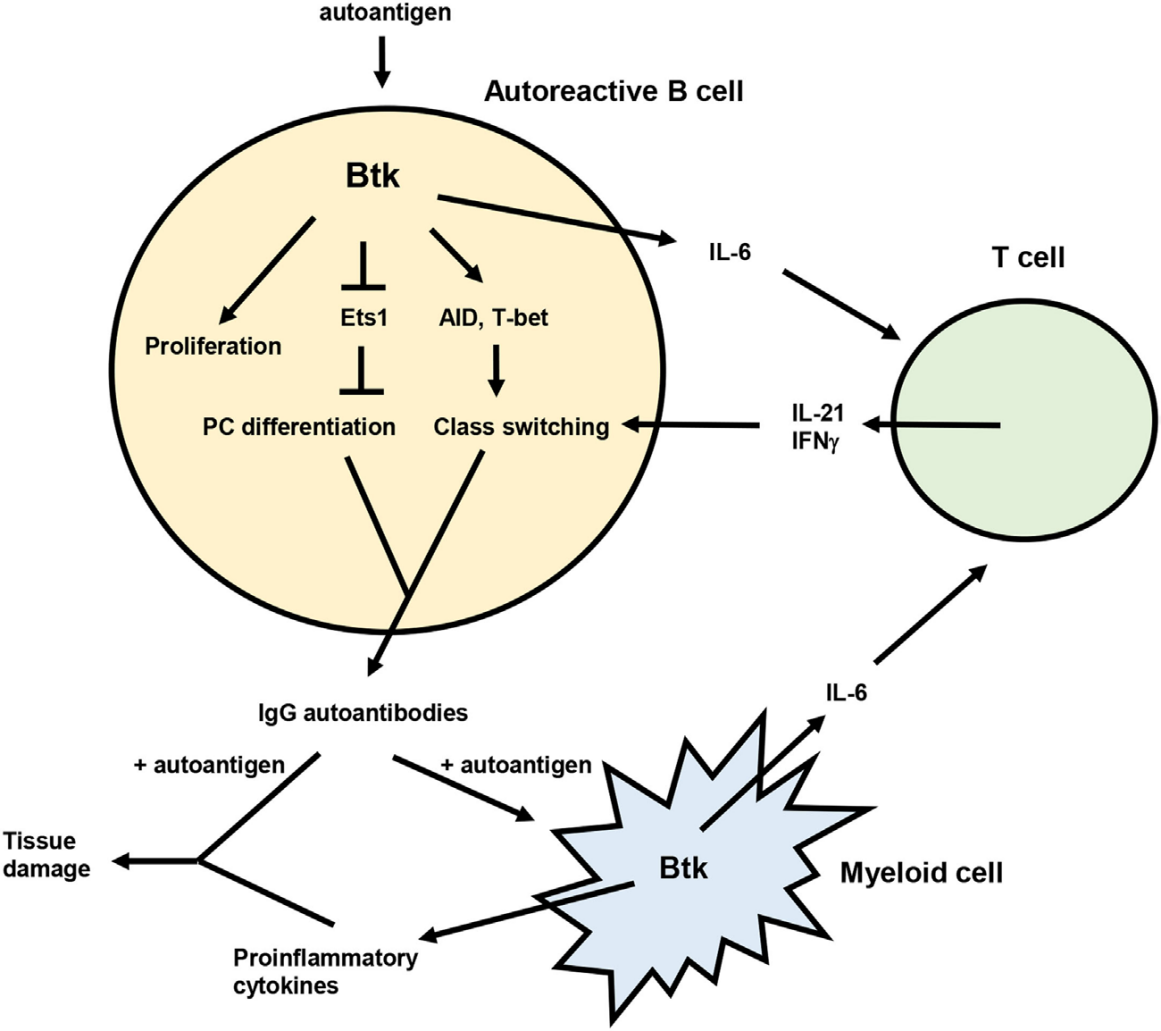
Model for the role of Bruton’s tyrosine kinase (Btk) in lupus pathogenesis. Btk acts in autoreactive B cells to promote proliferation, plasma cell (PC) differentiation, and class switching, resulting in the production of pathogenic IgG autoantibodies. IgG autoantibody production is also facilitated by the ability of Btk to enhance IL-6 expression from both B and myeloid cells. IL-6 then acts on T cells to promote differentiation of Tfh cells and IFNγ producing T cells, which in turn contribute to autoreactive B cell class switching *via* IL-21 and IFNγ. IgG autoantibodies produced in a Btk-dependent manner can then form immune complexes with autoantigen that deposit in tissues and induce inflammation and damage. These immune complexes can also activate myeloid cells, likely in a Btk-dependent manner, to produce inflammatory mediators that also damage tissues.

Genetic studies demonstrate multiple roles for Btk in the development of murine lupus (Figure 1), including promoting the activation, differentiation, and class switching of autoreactive B cells. Btk inhibitors are effective at reducing autoantibodies and/or autoimmune symptoms in mouse lupus models and may act in both B and myeloid cells to exert these effects. In humans, several components of Btk signaling pathways are altered in B cells from lupus patients, and Btk expression and activation is elevated in B cells from other autoimmune diseases. Btk has dose-dependent effects on B cell activation and autoantibody production as illustrated by the phenotypes of Btk^lo^ and Btk-overexpressing mice. Such a rheostat effect of Btk (150) is supported by recent structural analysis indicating that Btk has graded degrees of activity (151), and suggests that partial inhibition of Btk may have significant functional consequences. Btk may thus be a useful therapeutic target for SLE. The Btk inhibitor ibrutinib is well tolerated and approved for treatment of several B cell malignancies (152, 153), and second generation, more specific inhibitors such as acalabrutinib are promising (154, 155). The use of these and other Btk inhibitors in B cell malignancy will be informative with respect to potential off-target and side effects (156, 157) that might be encountered in the context of autoimmune disease.

## Author Contributions

The author confirms being the sole contributor of this work and approved it for publication.

## Conflict of Interest Statement

The author declares that the research was conducted in the absence of any commercial or financial relationships that could be construed as a potential conflict of interest. The reviewer ET and handling editor declared their shared affiliation.
